# Endoscopic retrieval of a huge gastric trichobezoar after thermally induced cutting with a hook knife

**DOI:** 10.1055/a-2462-1897

**Published:** 2024-12-03

**Authors:** Ting Wei, Deliang Li, Saif Ullah, Dan Liu, Qingfen Zheng, Bingrong Liu

**Affiliations:** 1 191599 Department of Gastroenterology and Hepatology, The First Affiliated Hospital of Zhengzhou University, Zhengzhou, China

Trichobezoars, composed of ingested hair, may lead to severe complications, including gastrointestinal obstruction, perforation, and gastric bleeding 1 2 3 . Herein, we report a case of successful endoscopic retrieval of a trichobezoar after thermally induced cutting with a hook knife.

A 5-year-old girl complained of severe abdominal pain for 8 hours. Physical examination revealed a nontender mass in the epigastric region. Abdominal computed tomography revealed a heterogeneous gastric mass, 9 cm in the long axis ( Fig. 1 **a** ). Endoscopy revealed a large trichobezoar mixed with chyme and mucus occupying nearly the whole gastric lumen ( Fig. 1 **b** ). After obtaining written consent from the patient’s guardians, we decided to perform endoscopic therapy ( Video 1 ).

**Fig. 1:**
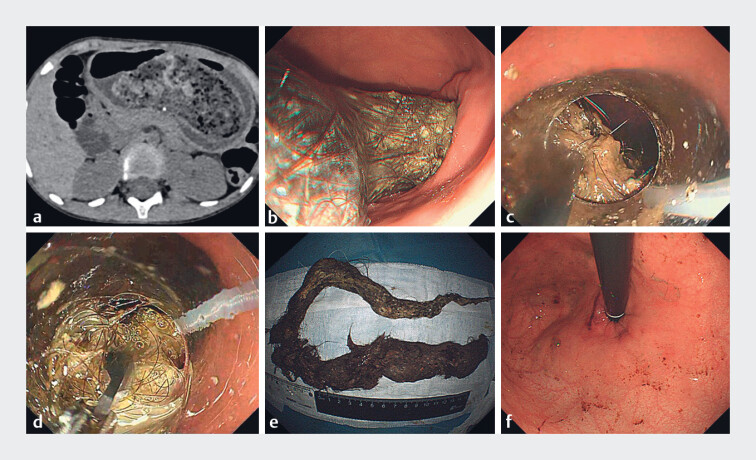
Imaging and removal of the gastric trichobezoar. **a** An abdominal computed tomography scan showed a heterogeneous gastric mass. **b** Endoscopy revealed a huge trichobezoar in the stomach. **c** A hook knife was used to break the huge trichobezoar, with externally controlled snare traction. **d** Foreign body forceps were used to retrieve the huge trichobezoar. **e** The trichobezoar. **f** The fundus after removal of the trichobezoar.

A novel endoscopic method for treating a large gastric trichobezoar: thermally induced cutting using a hook knife.Video 1

For the main procedure, a single-channel endoscope with a transparent cap and a snare attached to the tip was introduced into the gastric cavity. Foreign body forceps were used to grasp a portion of the trichobezoar and the snare was released to encircle this portion. Then, using externally controlled snare traction, a hook knife (KD-620LR; Olympus, Tokyo, Japan) with an electrosurgical generator (VIO300D; Erbe Elektromedizin GmbH, Tübingen, Germany), employing the Endocut Q mode, effect 3, with a cutting power of 60 W, was used to cut the first portion of the trichobezoar along its long axis into five fragments, with an interval of approximately 2.5 cm between each cut ( Fig. 1 **c** ). Fragments were then extracted using foreign body forceps and a snare ( Fig. 1 **d** ). The overall duration of the procedure was about 1.5 hours.

No significant damage was observed in the esophagus or cardia after complete removal of the trichobezoar ( Fig. 1 **e, f** ). The patient resumed a normal diet 3 days later and was discharged.

This is the first time that we have used thermally induced cutting with a hook knife to treat a huge trichobezoar. We demonstrated that this method could be feasible and effective.

Endoscopy_UCTN_Code_TTT_1AO_2AL

## Notes

Endoscopy E-Videos https://eref.thieme.de/e-videos
*E-Videos* is an open access online section of the journal *Endoscopy* , reporting on interesting cases and new techniques in gastroenterological endoscopy. All papers include a high-quality video and are published with a Creative Commons CC-BY license. Endoscopy E-Videos qualify for HINARI discounts and waivers and eligibility is automatically checked during the submission process. We grant 100% waivers to articles whose corresponding authors are based in Group A countries and 50% waivers to those who are based in Group B countries as classified by Research4Life (see: https://www.research4life.org/access/eligibility/ ). This section has its own submission website at https://mc.manuscriptcentral.com/e-videos .
